# An extremely rare case of giant oncocytic adenolipoma of the parotid gland

**DOI:** 10.1002/ccr3.3151

**Published:** 2020-07-19

**Authors:** Dipesh Shakya, Ajit Nepal

**Affiliations:** ^1^ Department of Otorhinolaryngology Civil Service Hospital Kathmandu Nepal; ^2^ Department of Otorhinolaryngology School of Medicine Patan Academy of Health Sciences Lalitpur Nepal

**Keywords:** adenolipoma, lipoadenoma, oncocyte, parotid

## Abstract

Oncocytic adenolipoma is a rare tumor to occur in the salivary gland, which can present as a giant neck mass. Until now, <20 such cases are reported. We report this rare case for surgeons to consider it as one of the differential diagnoses.

## Graphical abstract

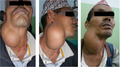

## 1 INTRODUCTION

Oncocytic adenolipoma is a rare tumor composed of adipose tissue and oncocytic epithelial cells in different proportions presenting in the salivary gland. Until now, <20 such cases are reported. We report another such rare case presented as a giant neck mass.

Lipomatous tumors are one of the commonly seen tumors of subcutaneous soft tissue. However, those originating from the salivary gland are extremely rare.^1^ It can be monophasic or biphasic. The monophasic tumor is more common than biphasic. Biphasic tumors consist of epithelial and the adipocytic component. Oncocytic adenolipoma or lipoadenoma is one of the rare biphasic lipomatous tumors which is composed of oncocytes admixed with mature adipocytes.^2^ It differs from the sialolipoma in which the epithelial part consists of normal looking serous acini.^3^ Only the case reports are published in the literature due to its rarity. In the recent fourth edition 2017 World Health Organization (WHO) histologic classification of tumors of salivary glands, sialolipoma has been included, but oncocytic adenolipoma is still not included.^4^, ^5^ We report a case of a patient with a giant painless mass in the neck with a history of more than 18 years of swelling and underwent excision. The case was followed up for 5 years postsurgery to rule out recurrence. The paper is presented to consider for this rare differential diagnosis for neck mass.

## 2 CASE PRESENTATION

A 46‐year‐old man, from remote and difficult to reach part of the country, came to our center with complaints of huge neck mass in the right side, almost occupying the whole of the right neck from levels 2, 3, 4, and 5. Informed consent was obtained from the patient for the case presentation. He gave a history of swelling, which was gradually progressive and painless for more than 18 years. As the patient had poor financial status and was from the remote part of the country, he presented very late after the onset of disease. Late presentation to the hospital is a common problem in developing countries. However recently, for the past 2 years, the size of the swelling had increased progressively than before. With the fear of cancer, the patient somehow managed to visit the hospital. There was no history of xerostomia, dysphagia, shortness of breath, facial deviation, change in sensation, fever, no intraoral discharge, loss of weight or change in appetite, and no known comorbidity.

**FIGURE 1:**
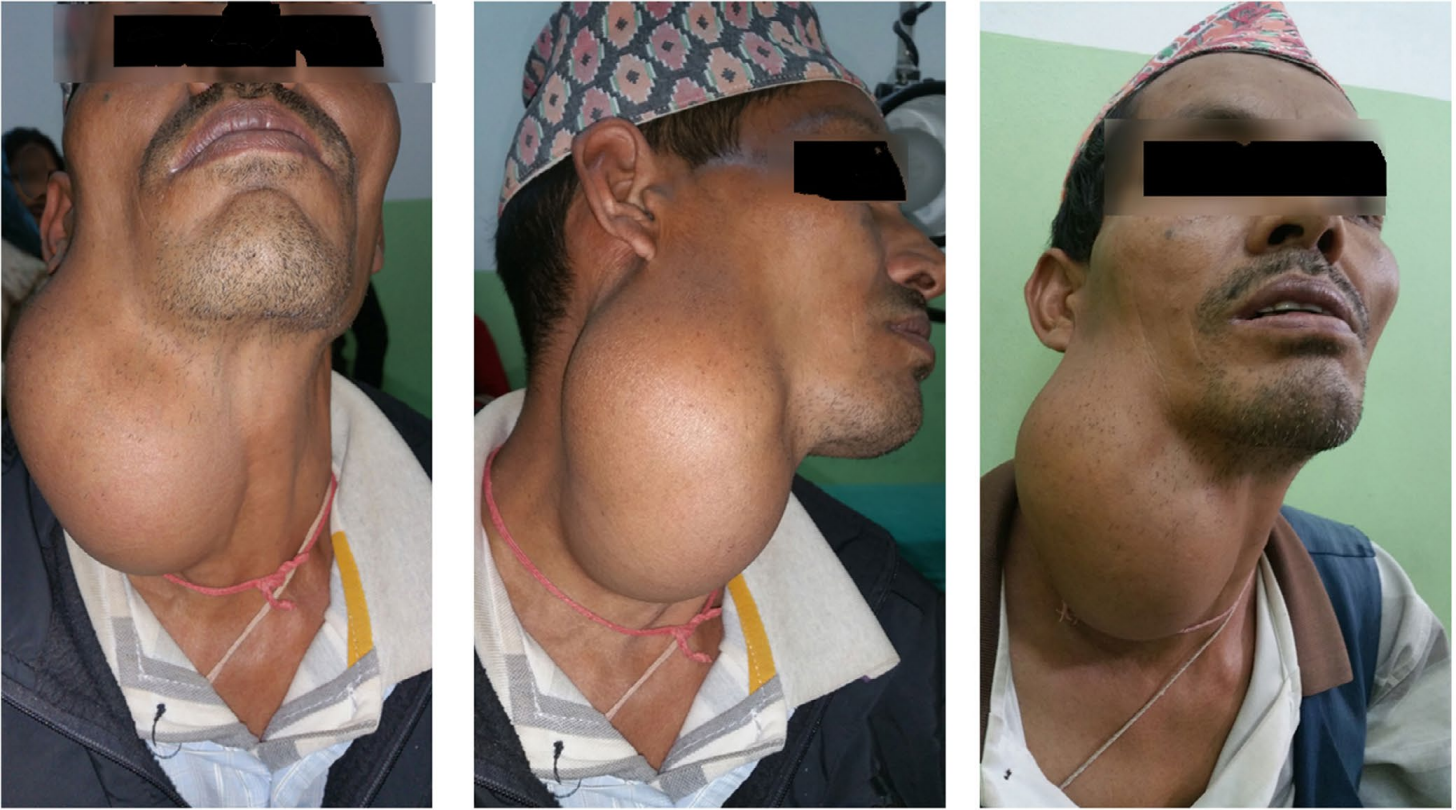
Giant mass seen in the neck in different views

On examination, the mass was well defined, multilobulated with a size of approximately 15 × 11 cm at the right side of neck, superiorly up to the level of ear lobule, inferiorly 4 cm above the clavicle, medially up to midline and posterolaterally occupying half of posterior triangle (Figure 1). The overlying skin was free, with no pain or tenderness on palpation, and the mass was firm in consistency, mobile, and multilobulated with a well‐defined border. There was no change in skin color, no sinuses, and no scar. Based on history and examination, a provisional diagnosis of soft‐tissue mass probably salivary gland origin was made. Fine‐needle aspiration cytology was done, which could not provide a definite opinion and just gave a suggestion of fat origin.

**FIGURE 2:**
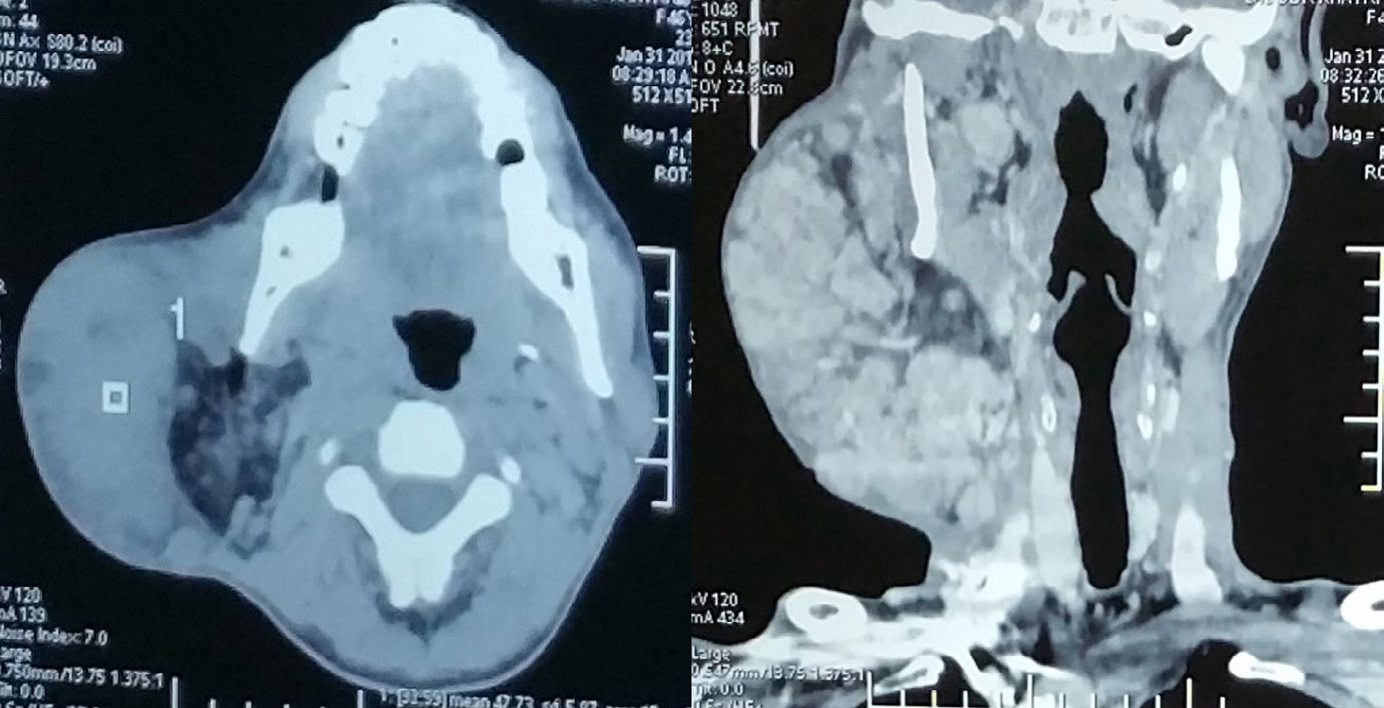
CT scan of the neck

CT scan was done, which reported as a huge lobulated mass measuring 15 × 10 × 6.5 cm in the right side of the neck and face. Radiologically, the mass contained enhancing solid areas on the periphery, which was supplied by large vessels and had fat components medially. No calcification or cystic areas noted and no significant lymph nodes. The lesion was abutting the parotid and submandibular gland. The CT reported lesion to be suggestive of the fat‐containing soft‐tissue tumor as angiolipoma with a differential of liposarcoma (Figure 2).

**FIGURE 3:**
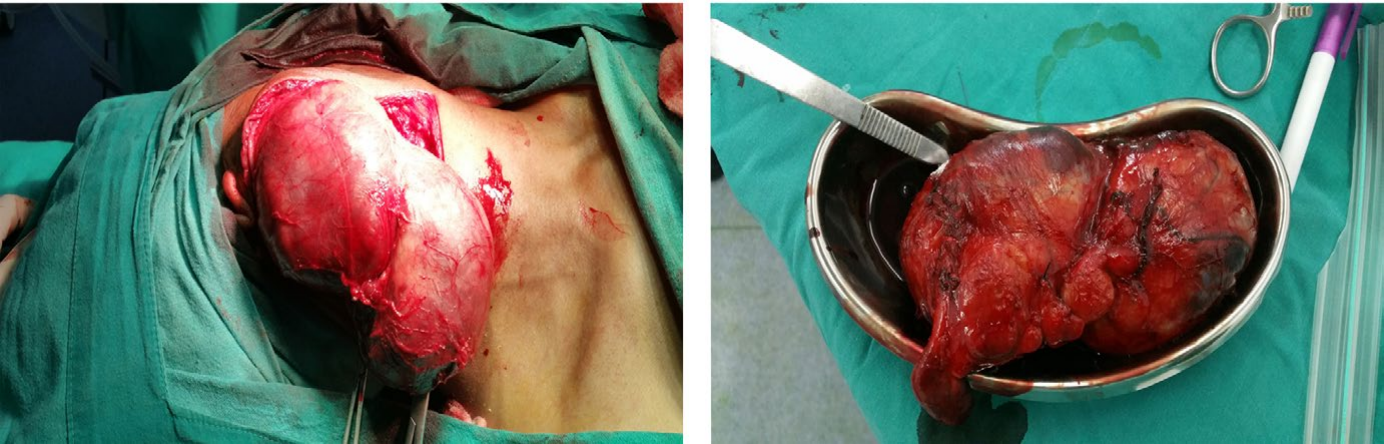
Surgical excision of the mass

**FIGURE 4:**
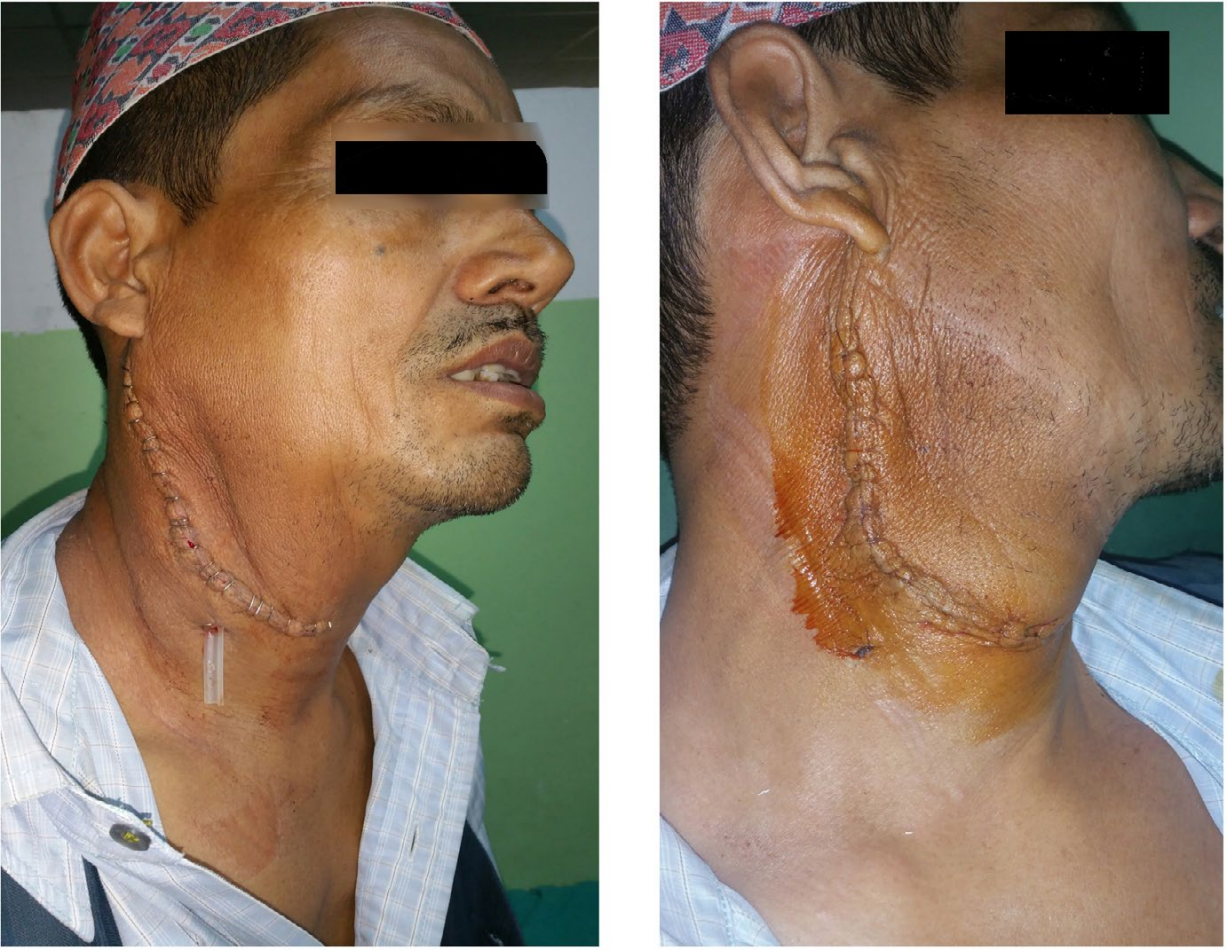
Postoperative wound status

The surgery was performed under general anesthesia. The vertical incision was given on the right side over the swelling. The subplatysmal flap was elevated. The capsule of the mass was dissected from all around the margin superiorly and inferiorly. The dissection was carried on securing the hemostasis. There were no findings suggestive of malignancy such as adhesions, friability of tissues, or invasion of surrounding tissues. Medially, the tissue was abutting the lower pole of the superficial lobe of the parotid gland. The tumor was seen arising from the part of the superficial lobe of the parotid gland. The mass was excised in toto and sent for histopathological examination. The drain was kept, and the surgical site was sutured (Figures 3 and 4).

Gross examination revealed a single piece of tissue comprising of two nodular tissues attached in the center by fibrofatty tissue measuring together 15 × 9.8 × 5 cm. The outer surface was nodular, brownish with congested vessels which were capsulated with pericapsular fat. Cut surface showed homogeneous brownish (mahogany brown) lobulated areas admixed with fatty tissue.

**FIGURE 5:**
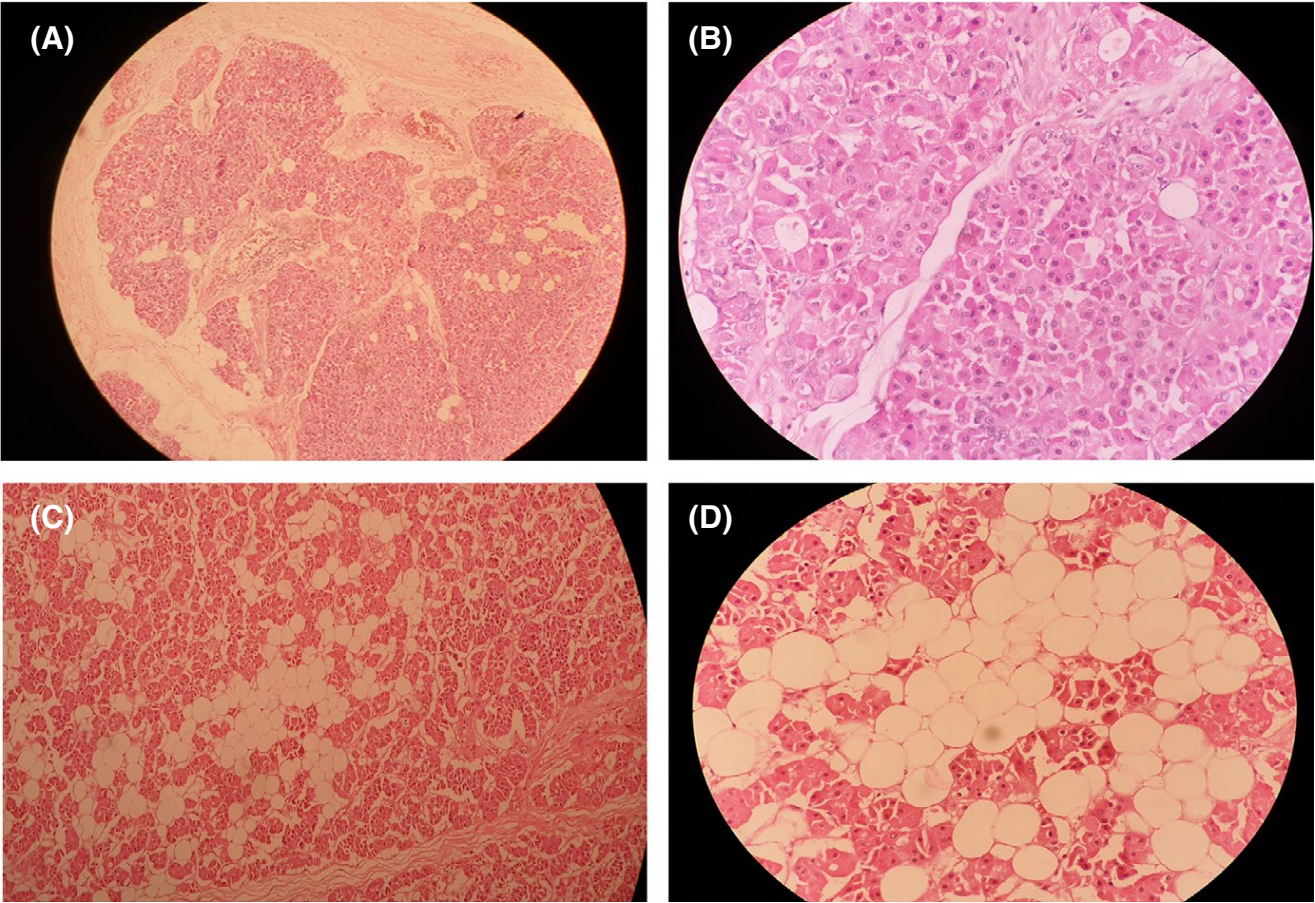
A, Biphasic tumor surrounded by a thin fibrous capsule (H&E, ×100). B, Oncocytes arranged in a solid pattern (H&E, ×400). C, Biphasic tumor seen with fibrous capsule, oncocytic epithelial component with adipose tissue component (H&E, ×200). D, High magnification of oncocytes distinct from adipocytes. (H&E, ×400)

Microscopic examination showed multiple lobules of tumor separated by thin fibrovascular septa with a fibrous capsule. The lobules composed of prominent oncocytes arranged in tubules, admixed with fatty tissue composed of mature adipocytes in varying proportions. Foci of squamous and sebaceous differentiation, chronic inflammatory cells, and stromal edema were evident as well. No features of malignancy noted (Figure 5). The final diagnosis of oncocytic adenolipoma of parotid gland origin was made.

## 3 DISCUSSION

Lipomatous tumors comprise 0.5% of salivary gland tumors, with the most common site being the parotid, followed by the submandibular gland.^2^ Histologically, its spectrum can range from pure lipomatous neoplasm like the one seen in the cutaneous site or can be admixed with epithelial component specific to salivary gland.^6^

Thus, it can be of monophasic (only lipomatous component) or biphasic (epithelial plus lipomatous component) histological type. Among the biphasic type, the epithelial component can be of oncocytic or nononcocytic.^2^ Oncocytic adenolipoma is an extremely rare benign tumor of salivary glands which consists of oncocytic epithelial components that are admixed with mature adipocytes in varying proportions.^7^ The main cytological feature distinctive of oncocytic lipoadenoma is the presence of adipose tissue fragments with a prominent lipoid background in addition to the oncocytic epithelial component.^8^ The first case of oncocytic adenolipoma of the submandibular gland was reported by Hirokawa et al only in 1998.^9^, ^10^ But, the first case of tumor originating from the parotid gland was reported by Kato et al in 2000.^11^ Subsequently, the second case from the parotid was published by Klieb et al in 2006.^12^ When Chi et al reported in 2015, only 18 such cases were reported in the literature.^13^ This rare condition has been published mainly as single case presentation in the literature under different names as oncocytic sialolipoma, oncocytic lipoadenoma, and adenolipoma.^6^

Most of the cases in the literature revealed that the oncocytic adenolipoma presented as a slowly growing, asymptomatic swelling most commonly from the parotid gland. The other areas where adenolipoma can be found include breast, thyroid, parathyroid, and skin.^14^ The duration of symptoms ranged from 15 days to 11 years.^15^ However, in our case, the history of onset was more than 18 years. Such a long duration and the size of the largest dimension of 15 cm has not been reported before. The biggest size reported was 14 cm of the parotid gland by Chahwala et al, which was 1 cm less than our case.^16^ The age of onset has been widely reported from 7 to 89 years old, but mostly seen in adults with a mean age of 57.7 years old.^17^ There are literature stating equal sex predilection to being slight male predominance of 2‐4:1.^6^ The treatment usually consists of excision with no risk of recurrence or aggressive behavior.^17^

The pathogenesis of the lesion is still elusive. There are different speculations regarding it. One theory given by Nagao stated it as entrapment of glandular component during lipomatous proliferation. He suggested it being not of neoplastic origin but as a type of lipoma rather than hamartomatous origin.^1^ However, Parente et al found the disorganized proliferation of neural and vascular structures by which they gave a theory of hamartomatous origin.^18^ The hypothesis of the hamartomatous nature given by Parente et al was negated by Akrish et al, who proposed another theory. Their theory suggested that it was the dysfunction of the salivary gland, leading to a modification of the normal gland function that leads to the tumor. This theory was supported by reasons such as prolonged history, lack of recurrence after excision, and histological features as ductal ectasia, periductal fibrosis, oncocytic metaplasia, and replacement of glandular tissue with mature adipocytes and chronic inflammatory cells.^19^ Ilie et al performed a molecular cytogenetic analysis of the tumor and showed a translocation t(12;14), resulting in a structural rearrangement of the region framing the HMGA2 gene at 12q14.3. This could have been the reason for cell proliferation. However, since the whole specimen was prepared for the cell culture, the cell origins for the tumor being adipocytes, or oncocytes, and/or basal cells were unknown.^20^

Clinically, the differential diagnosis of the present case with a huge lesion includes varieties of the neck mass, including all benign lesions of the salivary gland. Radiological investigation of CT scan helped us to narrow down the differential to the fat‐containing lesion as lipoma to lipoadenoma. Except for the huge size of the mass, radiologically the lesion was not suggestive of being malignant. However, FNA cytology was not found to be helpful for us. Histologically, oncocytic adenolipoma consists of circumscribed biphasic tumor surrounded by a thin fibrous capsule in which the adipose tissue intermingles with the oncocytic part. The epithelial component is formed only by the oncocytes with no ductal or acinar component, which is usually seen in sialolipoma.^3^ The differential diagnosis of oncocytic adenolipoma includes all other lesions containing oncocytes, such as oncocytoma, oncocytic carcinoma, Warthin's tumor, and oncocytic cystadenoma, as well as oncocytic variants of pleomorphic adenoma, myoepithelioma, mucoepidermoid carcinoma, and epithelial‐myoepithelial carcinoma.^6^, ^14^, ^17^ The excisional surgery was done as suggested by previous literature.^2^, ^10^, ^13^, ^15^ We followed the patient for 5 years, and until now, no recurrence of the lesion was noted. The present case is reported with the aim to make aware of the rare possibility of this extremely rare lipomatous lesion of the salivary gland in a patient presenting with a giant neck mass. We also aimed to confirm the long‐term disease‐free interval of 5 years after the surgical resection in this lesion.

## 4 CONCLUSION

Oncocytic adenolipoma is a unique, extremely rare salivary gland neoplasm that one needs to be aware of and should also be regarded as one of the differential diagnoses in the patient presenting with any giant neck mass. Given the rarity, we describe this case to generate awareness and add to the few reported cases in the literature.

## CONFLICT OF INTEREST

None declared.

## AUTHOR CONTRIBUTIONS

DS: involved in conceptualization and preparation of manuscripts. AN: critically reviewed the manuscript.
